# Evaluation of Apixaban standard dosing in underweight patients with non-valvular atrial fibrillation: a retrospective cohort study

**DOI:** 10.1186/s12959-024-00613-8

**Published:** 2024-05-22

**Authors:** Khalid Al Sulaiman, Ohoud Aljuhani, Hadeel Alkofide, Manal A. Aljohani, Hisham A. Badreldin, Mahasen Al Harbi, Ghalia Aquil, Raghad Alhajaji, Rahaf A. Alqahtani, Alaa Babonji, Maha Altuwayr, Asma A. Alshehri, Mashael Alfaifi, Abdullah F. Alharthi, Mohammed Alzahrani, Tareq Al Sulaiman, Nasser Alqahtani, Walaa A. Alshahrani, Abdulmalik Al Katheri, Abdulkareem M. Albekairy

**Affiliations:** 1 Pharmaceutical Care Department, King Abdulaziz Medical City, Riyadh, Saudi Arabia; 2 College of Pharmacy, Ministry of National Guard Health Affairs (MNGHA), King Saud bin Abdulaziz University for Health Sciences, PO Box 22490, Riyadh, 11426 Saudi Arabia; 3 King Abdullah International Medical Research Center, Riyadh, Saudi Arabia; 4 Saudi Critical Care Pharmacy Research (SCAPE) Platform, Riyadh, Saudi Arabia; 5 Department of Pharmacy Practice, Faculty of Pharmacy, King Abdulaziz University, Jeddah, Saudi Arabia; 6 Department of Clinical Pharmacy, College of Pharmacy, King Saud University, Riyadh, Saudi Arabia; 7 Pharmaceutical Care Department, King Abdulaziz Medical City, Jeddah, Saudi Arabia; 8 Pharmaceutical Care Department, King Faisal Specialist Hospital and Research Center, Jeddah, Saudi Arabia; 9 Public Health Department, Makkah Health Affairs, Makkah, Saudi Arabia; 10 Primary Health Department, Makkah Health Cluster, Makkah, Saudi Arabia; 11 Community Pharmacy, Makkah, Saudi Arabia; 12 Pharmaceutical care departments, Prince Sultan Military Medical City, Riyadh, Saudi Arabia; 13 Pharmaceutical Services Administration, King Saud Medical City, Riyadh, Saudi Arabia; 14 College of Pharmacy, Shaqra University, Shaqra, Saudi Arabia; 15 Department of Orthopedic Surgery, Imam Abdulrahman Al Faisal Hospital, Riyadh, Saudi Arabia; 16 School of Pharmacy, Alfaisal University, Riyadh, Saudi Arabia; 17 Saudi Society for Multidisciplinary Research Development and Education (SCAPE Society), Riyadh, Saudi Arabia; 18 Drug Regulation Research Unit, College of Pharmacy, King Saud University, Riyadh, Saudi Arabia

**Keywords:** Apixaban, Underweight, Weight < 50 kg, BMI < 25, Atrial fibrillation, Stroke, Thrombosis, Bleeding

## Abstract

**Background:**

Recent guidelines recommend using direct oral anticoagulants (DOACs) as first-line agents in patients with non-valvular atrial fibrillation (NVAF). Research is currently investigating the use of Apixaban in underweight patients, with some results suggesting altered pharmacokinetics, decreased drug absorption, and potential overdosing in this population. This study examined the effectiveness and safety of standard Apixaban dosing in adult patients with atrial NVAF weighing less than 50 kg.

**Methods:**

This is a retrospective cohort study conducted at King Abdulaziz Medical City (KAMC); adult patients with a body mass index (BMI) below 25 who received a standard dose of Apixaban (5 mg twice daily) were categorized into two sub-cohorts based on their weight at the time of Apixaban initiation. Underweight was defined as patients weighing ≤ 50 kg, while the control group (Normal weight) comprised patients weighing > 50 kg. We followed the patients for at least one year after Apixaban initiation. The study’s primary outcome was the incidence of stroke events, while secondary outcomes included bleeding (major or minor), thrombosis, and venous thromboembolism (VTE). Propensity score (PS) matching with a 1:1 ratio was used based on predefined criteria and regression model was utilized as appropriate.

**Results:**

A total of 1,433 patients were screened; of those, 277 were included according to the eligibility criteria. The incidence of stroke events was lower in the underweight than in the normal weight group at crude analysis (0% vs. 9.1%) p-value = 0.06), as well in regression analysis (OR (95%CI): 0.08 (0.001, 0.76), p-value = 0.002). On the other hand, there were no statistically significant differences between the two groups in the odds of major and minor bleeding (OR (95%CI): 0.39 (0.07, 2.03), p-value = 0.26 and OR (95%CI): 1.27 (0.56, 2.84), p-value = 0.40, respectively).

**Conclusion:**

This exploratory study revealed that underweight patients with NVAF who received standard doses of Apixaban had fewer stroke events compared to normal-weight patients, without statistically significant differences in bleeding events. To confirm these findings, further randomized controlled trials with larger sample sizes and longer observation durations are required.

**Supplementary Information:**

The online version contains supplementary material available at 10.1186/s12959-024-00613-8.

## Introduction

Nonvalvular atrial fibrillation (NVAF), characterized by the absence of moderate-to-severe mitral stenosis or a mechanical heart valve, is a notably prevalent type of cardiac arrhythmia commonly encountered in clinical practice. This condition is increasingly being recognized as a global health concern, resulting in a significant increase in healthcare expenditure, as well as increased morbidity and mortality rates [1]. In a community-based study, NVAF cases were estimated to reach up to 16 million cases by 2050, which highlights the need for effective management strategies [2].

Direct-acting anticoagulants (DOACs) are mostly favored for their predictable pharmacodynamic and pharmacokinetic properties which don’t necessitate drug level monitoring as recognized by manufacturers’ reference. They have been recognized as the primary therapeutic approach in mitigating the risk of stroke and thromboembolic events in patients with NVAF [3]. They have demonstrated favorable effectiveness and safety that is non-inferior to the use of vitamin K antagonists (VKA) [4, 5]. 

The therapeutic effectiveness of DOACs is intricately linked to their plasma concentration levels, which are, in turn, influenced by the volume of their body distribution. This relationship suggests that body weight extremes could potentially impact the safety and effectiveness of these drugs. However, the lack of specific dosing guidelines for DOACs in patients with extreme body weight largely due to the limited inclusion in randomized controlled trials involving DOACs is an emergent issue [4, 5, 7, 8]. A substantial number of patients with NVAF have been enrolled in prior landmark trials assessing the comparative effectiveness of VKAs versus DOACs. However, there appears to be a noticeable deficiency in the representation of NVAF patients classified as underweight (UW), with a Body Mass Index (BMI) less than 18.5 kg/m^2^, within these clinical trials [9]. 

Previous pharmacokinetic studies have indicated a potential risk of suboptimal dosing for DOACs in patients with significant variations in body weight. Specifically, morbidly obese patients may be at a higher risk of receiving an insufficient dose, thereby reducing the treatment effectiveness. Conversely, UW patients are at an increased likelihood of excessive dosage, and potentially prone to develop adverse events. This highlights the need for personalized dosing strategies that consider individual patient characteristics, such as body weight, to ensure optimal therapeutic outcomes [10, 11]. The standard recommended dosage for Apixaban, is 5 mg administered orally twice daily [6]. However, a reduced dosage of 2.5 mg twice daily is suggested for patients who meet at least two of the following parameters: age over 80 years, body weight under 60 kg, or serum creatinine level equal to or greater than 1.5 mg/dL (133 micromole/L) [6]. Additionally, the lower dose is recommended if the patient’s creatinine clearance (CrCl) ranges between 15 and 30 mL/min [3].

Current available evidence on NVAF patients with UW presents contradictory results [12–17]. Data derived from a meta-analysis by Grymonprez et al. demonstrated a significant higher incidence of stroke or thromboembolic events and mortality risks in UW NVAF populations. Though, no significant concern in terms of bleeding reports [14]. While a real-world registry analysis and another retrospective cohort study demonstrated a greater risk of bleeding [13, 16]. Conversely, the post hoc analysis of the ARISTOTLE trial in addition to multiple trials demonstrated that UW had no effects on the effectiveness and safety of Apixaban with no increased risk of bleeding nor thrombotic events [12, 13, 17–22]. 

The inconsistency of these results in addition to the lack of clinical evidence, raises a concern in terms of effectiveness and safety of using Apixaban in such a group of patients, and whether they should be on the standard dose, or a lower dose is needed for adjustment. Therefore, this exploratory cohort study aims to compare the effectiveness and safety of the standard dose of Apixaban being prescribed for NVAF in UW patients.

## Methods

### Study design

This retrospective cohort study was exploratory in nature that included adult patients with a body mass index (BMI) less than 25 who received a standard dose of Apixaban (5 mg twice daily) regardless of their CHA_2_DS_2_-VAS_2_c score at King Abdulaziz Medical City (Riyadh) between January 01st, 2016, and December 31st, 2019. Moreover, we implemented a categorization strategy in the whole cohort by based on the actual body weight of study participants at the time of Apixaban initiation. Patients were divided into two sub-cohorts: patients with a body weight of 50 kg or less (considered underweight) and a control group with a body weight exceeding 50 kg (normal weight) [6, 10]. Patients were then followed for a minimum of one year after Apixaban initiation. The Institutional Review Board at King Abdullah International Medical Research Center (KAIMRC) approved the study in June 2023 (Ref. #NRC23R-319-05). Informed consent from the study patients was waived due to the retrospective observational nature of the study.

### Study participants

During the study period, adult patients aged 18 years or above who had been prescribed Apixaban for NAAF were screened for eligibility. However, the study excluded patients who had confirmed diagnoses of liver cirrhosis Child C, APLS/SLE, mechanical valve, and patients who had a BMI above 25 at the time of Apixaban initiation. Moreover, patients who are known to have non-adherence history as indicated by their medical records, using Apixaban with a dose of ≤ 5 or > 10 mg/day, or those with incomplete data/lab results were also excluded.

### Study setting

This study was conducted in the King Abdulaziz Medical City located in Saudi Arabia, a tertiary-care academic referral hospital. King Abdulaziz Medical City is one of the leading medical facilities with a capacity of 1,601 beds. This medical city offers a wide range of services from primary health care needs to highly specialized tertiary care to the health needs of National Guard staff and employees and their families, providing an all-encompassing spectrum of healthcare services. The center is equipped with cutting-edge technology and staffed by highly qualified healthcare professionals.

### Data collection

The data for each patient was gathered and extracted from the electronic hospital’s record system, BESTCare 2.0 A, and compiled into an Excel spreadsheet for analysis. We recorded various information, including patients’ demographic details, comorbidities, vital signs, and laboratory test results. Risk scores for stroke and bleeding, namely the CHA_2_DS_2_-VAS_2_c and HAS-BLED scores, were also documented at the time of Apixaban initiation. Additional laboratory tests encompassed the coagulation profile, liver and renal function tests, and complete blood count (including hemoglobin and platelet levels). Information on concurrently using antiplatelet medications and gastrointestinal (GI) prophylaxis was also documented. Furthermore, we collected data on various clinical events, including the incidence of stroke (either ischemic or hemorrhagic), pulmonary embolism (PE), deep vein thrombosis (DVT), and upper or lower GI bleeding (verified through medical documentation or procedures like upper endoscopy or colonoscopy). The presence of left ventricular thrombus, as confirmed by an echocardiogram, while on Apixaban was also recorded.

### Outcomes

In this exploratory cohort study, the aim was to compare the effectiveness and safety of the standard dosing of Apixaban (5 mg twice daily) in underweight patients (body weight ≤ 50 kg) with patients in the normal weight group, all of whom have NVAF. The primary outcome was the stroke events. In contrast, the secondary outcomes were bleeding (major and minor), all Thrombosis causes, DVT and PE, after Apixaban initiation (Outcomes Definition – Supplementary File 1) [23, 24] [30–31].

### Statistical analysis

Descriptive analyses were presented as mean ± SD and median with interquartile range (IQR) for continuous data, and numbers and percentages for categorical data. The characteristics of patients in the normal weight group were compared to those in the underweight group using the t-test and Mann-Whitney U test for continuous data and the Chi-Square or Fisher exact test for categorical data as appropriate.

Propensity score matching was used to balance the differences in baseline characteristics between the normal and underweight groups. Propensity scores estimating the likelihood of being underweight versus normal weight were calculated using logistic regression, incorporating age, gender, and CHA2DS2-VASc Score as predictors. We then matched normal weight individuals to their underweight counterparts at a 1:1 ratio using a greedy nearest neighbor approach, ensuring each pair consisted of one normal weight and one underweight patient. Standardized difference was used to assess the balance of covariates after matching, and a standardized difference < 10% was considered acceptable.

Firth’s logistic regression was used to examine the correlation between normal weight (considered as the reference) and underweight in relation to study outcomes (R package logistf V 1.24). Firth’s logistic regression addresses estimation issues related to low event rates and complete separation. When using the pre-matched cohort, all models included the weight status variable and were adjusted for variables that were associated with the study outcomes in univariable analyses. In the post-matched cohort, regression analysis was done by considering the PS score as one of the covariates in the model. The odds ratios (OR) or estimates with 95% confidence intervals (CIs) were reported as appropriate. No imputation was made for missing data, as the cohort of patients in our study was not derived from random selection. A P value < 0.05 was considered to be statistically significant. All statistical analyses were performed using R statistical software version 4.3.1.

## Results

### Demographic and clinical characteristics

**Fig. 1:**
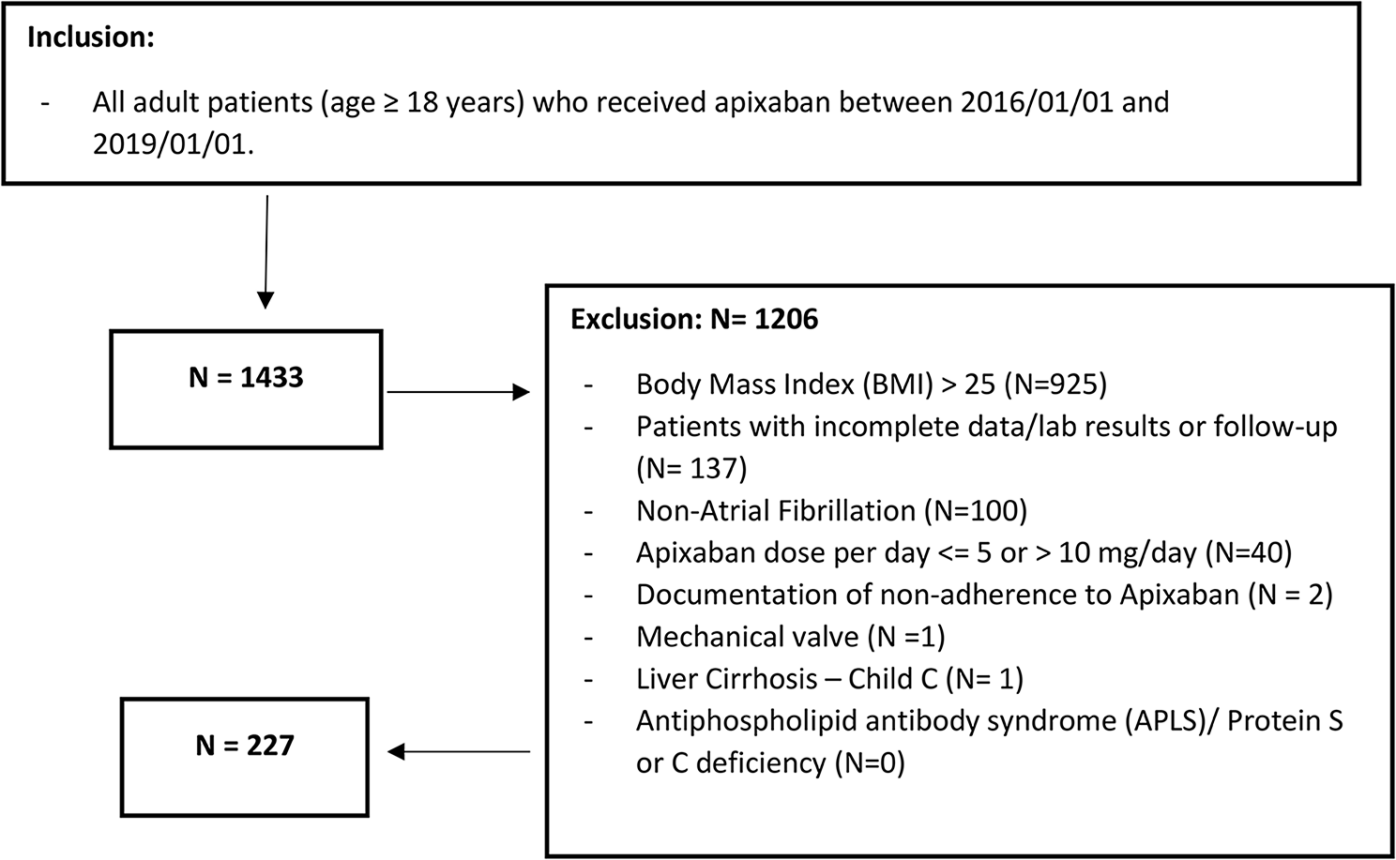
Flowchart showing atrial fibrillation patients who received apixaban

A total of 1,433 patients were screened for participation in this study. Among them, 277 patients met the eligibility criteria and were included in the analysis, as shown in Fig. 1. The baseline characteristics of the study population before and after propensity score (PS) matching are presented in Table 1. Of the included patients, 172 belonged to the normal weight group, whereas 55 individuals were underweight. Prior to PS matching, male gender and COPD were more predominant in the normal-weight group compared with underweight patients. In addition, blood glucose levels and Alanine transaminase (ALT) were higher in the normal weight group. On the other hand, CHA_2_DS_2_-VAS_2_c Score was higher in patients who were categorized as underweight. However, the two groups became comparable after utilizing PS (1:1 ratio) matching based on the predefined criteria (Table 1).

**Table 1:** Baseline characteristics

	Baseline Characteristics- Pre-Matching				Baseline Characteristics- Post-Matching			
	Overall (N = 227)	Normal Weight (N = 172)	Underweight (N = 55)	P-value	Overall (N = 110)	Normal Weight (N = 55)	Underweight (N = 55)	P-value
Male, n(%)	142 (62.6%)	123 (71.5%)	19 (34.5%)	< 0.001	38 (34.5%)	19 (34.5%)	19 (34.5%)	0.999
**Age, Years**								
Mean (SD)	76.0 (13.2)	75.7 (13.1)	76.9 (13.6)	0.583	76.9 (13.7)	76.9 (13.8)	76.9 (13.6)	0.983
Median [Min, Max]	78 [29, 105]	79 [29, 101]	78. [39, 105]		79.5 [29, 105]	81 [29, 101]	78 [39, 105]	
**Age > 75 years**	139 (61.2%)	105 (61.0%)	34 (61.8%)	0.999	71 (64.5%)	37 (67.3%)	34 (61.8%)	0.690
**Age, 65–74, years**	49 (21.6%)	36 (20.9%)	13 (23.6%)	0.813	23 (20.9%)	10 (18.2%)	13 (23.6%)	0.639
**Actual Weight, Kg**								
Mean (SD)	57.4 (9.3)	61.2 (6.9)	45.6 (4.1)	< 0.001	52.4 (8.4)	59.2 (5.49)	45.6 (4.1)	< 0.001
Median [Min, Max]	57 [36, 81]	60 [51, 81]	47 [36, 50]		50.5 [36, 72]	58.0 [51, 72]	47 [36, 50]	
**BMI, Kg/m2**								
Mean (SD)	21.9 (2.44)	22.7 (1.82)	19.5 (2.53)	< 0.001	21.2 (2.73)	22.9 (1.60)	19.5 (2.53)	< 0.001
Median [Min, Max]	22 [12, 25]	23 [17, 25]	20 [12, 24]		22 [12, 25]	23 [17, 25]	20 [12, 24]	
**Comorbidity, n(%)**								
**Diabetes Mellitus**	154 (67.8%)	118 (68.6%)	36 (65.5%)	0.788	74 (67.3%)	38 (69.1%)	36 (65.5%)	0.839
**Hypertension**	180 (79.3%)	134 (77.9%)	46 (83.6%)	0.471	91 (82.7%)	45 (81.8%)	46 (83.6%)	0.998
**Hypothyroidism**	36 (15.9%)	26 (15.1%)	10 (18.2%)	0.742	20 (18.2%)	10 (18.2%)	10 (18.2%)	0.999
**Dyslipidemia**	100 (44.1%)	76 (44.2%)	24 (43.6%)	0.999	48 (43.6%)	24 (43.6%)	24 (43.6%)	0.989
**CVA, Stroke**	73 (32.2%)	55 (32.0%)	18 (32.7%)	0.999	35 (31.8%)	17 (30.9%)	18 (32.7%)	0.989
**VTE**	22 (9.7%)	14 (8.1%)	8 (14.5%)	0.256	14 (12.7%)	6 (10.9%)	8 (14.5%)	0.775
**CHF**	22 (9.7%)	14 (8.1%)	8 (14.5%)	0.918	55 (50.0%)	27 (49.1%)	28 (50.9%)	0.999
**IHD**	75 (33.0%)	61 (35.5%)	14 (25.5%)	0.227	31 (28.2%)	17 (30.9%)	14 (25.5%)	0.672
**ACS**	17 (7.5%)	14 (8.1%)	3 (5.5%)	0.716	9 (8.2%)	6 (10.9%)	3 (5.5%)	0.487
**Vascular Disease**	2 (0.9%)	2 (1.2%)	0 (0%)	0.99	2 (1.8%)	2 (3.6%)	0 (0%)	0.475
**CABG**	19 (8.4%)	15 (8.7%)	4 (7.3%)	0.954	9 (8.2%)	5 (9.1%)	4 (7.3%)	0.999
**PCI**	25 (11.0%)	22 (12.8%)	3 (5.5%)	0.206	10 (9.1%)	7 (12.7%)	3 (5.5%)	0.32
**CKD**	54 (23.8%)	39 (22.7%)	15 (27.3%)	0.606	25 (22.7%)	10 (18.2%)	15 (27.3%)	0.363
**Liver disease**	11 (4.8%)	8 (4.7%)	3 (5.5%)	0.998	7 (6.4%)	4 (7.3%)	3 (5.5%)	0.999
**History of Major Bleeding**	1 (0.4%)	1 (0.6%)	0 (0%)	0.999	0 (0%)	0 (0%)	0 (0%)	
**Medication predisposing to bleed**	31 (13.7%)	25 (14.5%)	6 (10.9%)	0.648	16 (14.5%)	10 (18.2%)	6 (10.9%)	0.417
**CHA2DS2 VASc Score**								
Mean (SD)	4.47 (1.7)	4.37 (1.7)	4.78 (1.6)	0.0998	4.8 (1.7)	4.8 (1.7)	4.8 (1.6)	0.863
Median [Min, Max]	5 [0, 8]	4 [0, 8]	5 [1, 8]		5 [0, 8]	5 [0, 8]	5 [1, 8]	
**HAS BLED Score**								
Mean (SD)	2.37 (1.1)	2.34 (1.1)	2.45 (0.9)	0.457	2.4 (1.0)	2.4 (1.1)	2.5 (0.9)	0.852
Median [Min, Max]	2 [0, 5]	2 [0, 5]	2 [0, 4]		2 [0, 5]	2 [0, 5]	2 [0, 4]	
**Concomitant antiplatelet use (e.g., Aspirin, Clopidogrel), n (%)**	106 (46.7%)	84 (48.8%)	22 (40.0%)	0.306	49 (44.5%)	27 (49.1%)	22 (40.0%)	0.443
**Serum creatinine, mmol/l**								
Mean (SD)	98.3 (76.6)	93.8 (40.0)	112 (138)	0.336	95.5 (100)	78.9 (24.3)	112 (138)	0.0849
Median [Min, Max]	82 [39, 841]	84 [47, 314]	74 [39, 841]		74 [39, 841]	74 [47, 148]	74 [39, 841]	
**eGFR**								
Mean (SD)	78.2 (29.8)	77.9 (26.8)	79.1 (38.1)	0.824	80.1 (32.2)	81.0 (25.5)	79.1 (38.1)	0.767
Median [Min, Max]	76 [6, 190]	76 [18, 155]	77.5 [6, 190]		79 [6, 190]	79 [35, 155]	77.5 [6, 190]	
**Hematocrit**								
Mean (SD)	0.4 (0.1)	0.4 (0.07)	0.4 (0.05)	0.016	0.4 (0.1)	0.4 (0.1)	0.4 (0.1)	0.841
Median [Min, Max]	0.4 [0.2, 0.5]	0.4 [0.2, 0.5]	0.4 [0.2, 0.5]		0.4 [0.2, 0.5]	0.4 [0.3, 0.5]	0.4 [0.2, 0.5]	
**Platelets count**								
Mean (SD)	249 (108)	252 (112)	242 (95.8)	0.51	246 (93.1)	251 (91.1)	242 (95.8)	0.605
Median [Min, Max]	233 [60, 698]	233 [60, 698]	230 [82, 477]		237 [82, 600]	241 [100, 600]	230 [82, 477]	
**ALT**								
Mean (SD)	33.2 (85.9)	36.5 (97.3)	22.0 (15.6)	0.0641	24.5 (19.9)	26.8 (23.1)	22.0 (15.6)	0.207
Median [Min, Max]	19 [2, 1210]	19.0 [2, 1210]	17.0 [5, 71]		17 [2, 112]	19 [2, 112]	17 [5, 71]	
**AST**								
Mean (SD)	36.2 (69.6)	38.5 (78.9)	28.6 (13.7)	0.126	27.7 (14.7)	26.9 (15.7)	28.6 (13.7)	0.562
Median [Min, Max]	23 [1, 712]	22 [1, 712]	24 [10, 72]		23.5 [1, 79]	22 [1, 79]	24 [10, 72]	
Bilirubin, Total								
Mean (SD)	14.1 (10.7)	14.1 (11.4)	14.0 (8.0)	0.976	13.2 (7.8)	12.5 (7.6)	14.0 (8.0)	0.309
Median [Min, Max]	11 [3.6, 98.9]	11 [3.6, 98.9]	11.3 [4.2, 43]		11 [3.9, 48.1]	10.9 [3.9, 48.1]	11.3 [4.2, 43]	

Comparison was done using unpaired t-test for continuous data, and chi-square or fisher-exact test for categorical data

Abbreviations: CVA; Cerebral Vascular Accident, VTE; Venous Thromboembolism, CHF; Congestive Heart Failure, IHD; Ischemic Heart Disease, ACS; Acute Coronary Syndrome, CABG; Coronary Artery Bypass Graft Surgery, PCI; Percutaneous Coronary Intervention, CKD; Chronic Kidney Disease, eGFR; Estimated Glomerular Filtration Rate, ALT; Alanine Transaminase, AST; Aspartate Aminotransferase

### Stroke and thrombosis

The rate of stroke events was higher in the normal weight group compared to underweight patients using standard dose of Apixaban (9.1% vs. 0%; p-value = 0.06). Using Firth’s logistic regression analysis, the odds of stroke events were statistically lower in patients belonging to the underweight group (OR 0.08, 95% CI 0.001, 0.76; P = 0.002). Moreover, all thrombosis causes were lower in the underweight group; however, it was not statistically significant (OR 0.55, 95% CI 0.15, 1.86; P = 0.34). Furthermore, venous thromboembolism (VTE) occurrences were similar between the two groups (OR 1.00, 95% CI 0.15, 6.69; P = 0.98). (Table 2).

**Table 2:** Primary and secondary outcomes among matched cohort of patients taking Apixaban for atrial fibrillation

Outcome, n(%)	Normal Weight Group^&^	Under Weight Group	P-value*	Odds ratio (OR) (95% CI)**	P-value**
Stroke	5 (9.1%)	0 (0%)	0.06	0.08 (0.001, 0.76)	0.002
VTE	2 (3.6%)	2 (3.6%)	0.99	1.00 (0.15, 6.69)	0.98
All Thrombosis causes	7 (12.7%)	4 (7.3%)	0.36	0.55 (0.15, 1.86)	0.34
Major Bleeding	2 (3.6%)	1 (1.8%)	0.99	0.59 (0.05, 4.57)	0.61
Minor Bleeding	8 (14.5%)	10 (18.2%)	0.78	1.29 (0.48, 3.56)	0.61

Abbreviations: VTE; Venous Thromboembolism

& Reference group

*P-value calculated using Chi Square or Fisher exact test

**Odds ratio calculated using Logistic Regression analysis or Firth’s Regression analysis (for low event rate outcome). These models adjusted for age, gender, and CHA_2_DS_2_-VAS_2_c Score

### Bleeding events

In crude analysis, major bleeding occurred in two patients in the normal weight group compared with one patient in the underweight group (3.6% vs. 1.8%; p-value = 0.99); however, it was not statistically significant (OR 0.59, 95% CI: 0.05, 4.57; P = 0.61). (Table 2). In contrast, minor bleeding events were slightly higher in the underweight patients (18.2% vs. 14.5%; p-value = 0.78); but were not statistically significant neither in crude or regression analyses (OR 1.29, 95% CI 0.48, 3.56; P = 0.61) (Table 2). Of importance, the HAS-BLED score, concomitant use of antiplatelet, and GI prophylaxis were not statistically significant between the two groups after PS matching (Table 1).

## Discussion

In this exploratory retrospective cohort study we found that the use of standard doses of apixaban in underweight patients with AF is associated with a significantly lower risk of stroke compared to normal weight patients. Moreover, all thrombosis events were none significantly lower in the underweight group. After adjusting for confounders, neither group had significantly greater risk for major bleeding or VTE occurrence.

Both the mean and median weights of patients exceeding 50 kg were below 60 kg. Specifically, the mean weight was 59.2 kg with a standard deviation of 5.49, while the median was 58 kg. This weight range is considered a critical threshold for dose adjustment, particularly when factoring in other variables influencing dosing. Our study’s baseline demographics revealed that over 70% of participants were elderly, potentially explaining this weight trend. Additionally, the presence of multiple comorbidities may contribute, as individuals with chronic conditions often exhibit lower weights compared to their healthier counterparts within the same age group.

We found that using the standard doses of Apixaban in underweight patients was more effective than utilizing the same dosage in normal-weight patients which is consistent with the previous published studies. A post hoc analysis of 1,985 patients (10.9%) in the ARISTOTLE study weighed 60 kg or less and found that the effectiveness and safety of Apixaban are consistent across all spectrums of weight [18]. Moreover, a retrospective cohort study assessing bleeding and thrombotic event rates for patients with AF who are prescribed Apixaban and have a low versus normal body weight (< 60 kg vs. 60 to 100 kg) demonstrated that there was no statistically significant difference in bleeding or thrombotic events between low and normal weight cohorts [17].

In terms of safety outcomes, neither group had a significantly higher risk of major bleeding or thrombosis after adjusting for confounders; however, interestingly, minor bleeding were numerically greater in the underweight patients group (OR 1.29, 95% CI 0.48, 3.56; P = 0.61) despite the fact that both groups had similar baseline distributions of bleeding risks including; medication predisposing to bleed, history of major bleed, CHA_2_DVAS_2_c score, HAS-BLED score, concomitant antiplatelet use, concomitant GI prophylaxis. In contrast, a retrospective analysis of patients receiving DOACs (Apixaban, Rivaroxaban, and Dabigatran) for AF revealed that the risk of major bleeding was considerably higher in the underweight group compared to the normal weight group [13]. These findings concurred with a meta-analysis based on four randomized trials and five observational studies that found no significant differences in the risk of major bleeding and intracranial bleeding among all the weight-based groups [14]. It’s interesting to note that most of these earlier trials compared warfarin with DOACs in general, but in our study, both groups consisted solely of individuals using Apixaban and were stratified according to the weight. The fact that the underweight patient group in our study had a numerically higher number of minor bleeding events could be attributed to the fact that this group was primarily composed of elderly patients > 75 years old (61.8%), heart failure patients (50%), cancer patients (3.6%), and patients with renal impairment (27.3%). Another crucial element to consider is how Apixaban’s pharmacokinetics fluctuate in people with extreme body weight [25].

The utilization of anti-factor Xa assays to gauge the anticoagulant impact of Apixaban in specific demographics, such as underweight individuals, remains limited, with only a handful of studies addressing this issue. [25–28]. Additionally, Anti-factor Xa assays for measuring the DOAC effect have limited availability in hospitals globally and was not available at our hospital. Additional data are necessary to determine the practicality of implementing this approach in a clinical setting.

This study is unique in that it examined standard doses of Apixaban in both groups stratified based on weight, as there aren’t any high-quality, randomized studies investigating the safety and efficacy of Apixaban in underweight individuals. Propensity matching was also employed to lessen the effect of any potential confounders that may have existed between the two groups. The present findings, however, contain some limitations of observational research, such as selection bias, and are based on results collected retrospectively from one site. Another limitation is the small percentage of underweight patients which may limit the interpretation of the clinical events. Furthermore, this study had a small sample size, limiting its power, yet its exploratory nature remains integral. Additionally, despite there being no clear guidelines for monitoring and its relationship to clinical outcomes, drug-level monitoring to check for Apixaban accumulation by measuring anti-factor Xa levels was not carried out in our cohort. We included patients who had undergone bariatric surgeries, acknowledging that such procedures can potentially affect the absorption of Apixaban, which could influence our study outcomes. Last, we were unable to evaluate the patients’ adherence Apixaban medication and any concurrent drug use or interactions or pharmacokinetics changes that might have an impact on the results of the study.

## Conclusion

This exploratory study showed that underweight patients with non-valvular AF who were administered standard doses of Apixaban experienced fewer stroke events compared to normal-weight patients without statistical significance differences regarding bleeding events. Further randomized controlled trials and pharmacokinetic/pharmacodynamic studies with larger sample size and extended follow-up are needed to confirm these findings.

### Electronic supplementary material

Below is the link to the electronic supplementary material.

Supplementary Material 1

## Data Availability

The datasets used and/or analyzed during the current study are available from the corresponding author on reasonable request.
