# Supplementary material for: Evaluation of Apixaban standard dosing in underweight patients with non-valvular atrial fibrillation: a retrospective cohort study
Source: Thromb J. 2024 May 22;22:43. doi: 10.1186/s12959-024-00613-8 (PMC11110266; doi:10.1186/s12959-024-00613-8)
Supplement: Supplementary file 1 — Supplementary Material 1 [file 12959_2024_613_MOESM1_ESM.docx]

**Outcome definitions**

Thrombotic events were identified using the International Classification of Diseases, Tenth Revision, Clinical Modification (ICD10-CM) code (i.e., stroke, pulmonary embolism, deep vein thrombosis), chart review documentation and/or radiology findings. All patients were monitored and observed for at least one year of Apixaban initiation.

Major bleeding was defined according to the ISTH as clinically overt bleeding associated with a fall in hemoglobin by ≥20 g/L, transfusion of ≥2 U packed red blood cells (PRBCs) or whole blood, retroperitoneal or intracranial bleeding, or fatal bleeding. All patients were monitored and observed for at least one year of Apixaban initiation (1,2).

Minor bleeding was defined according to the ISTH definition as any sign or symptom of bleeding that does not fit the criteria for the ISTH definition of major bleeding, but does meet at least one of the following criteria: requiring medical intervention by a healthcare professional, bleeding leading to hospitalization or increased level of care or prompting a face to face evaluation. All patients were monitored and observed for at least one year of Apixaban initiation (1,2).

**References**

1. Schulman S, Kearon C. Definition of major bleeding in clinical investigations of antihemostatic medicinal products in non-surgical patients. J Thromb Haemost. 2005 Apr;3(4):692–4.

2. Kaatz S, Ahmad D, Spyropoulos AC, Schulman S. Definition of clinically relevant non-major bleeding in studies of anticoagulants in atrial fibrillation and venous thromboembolic disease in non-surgical patients: Communication from the SSC of the ISTH. J Thromb Haemost. 2015 Nov 1;13(11):2119–26.
